# Brote de *tinea capitis* tricofítica en un grupo de niños escolares en un área rural del departamento del Cauca, Colombia

**DOI:** 10.7705/biomedica.6793

**Published:** 2023-08-31

**Authors:** Fabiola Eugenia González, José Alejandro Rodríguez, Lina María Muñoz, Giovanny Apráez, Luis Reinel Vásquez

**Affiliations:** 1 Facultad Ciencias de la Salud, Universidad del Cauca, Popayán, Colombia Universidad del Cauca Universidad del Cauca Popayán Colombia; 2 Secretaría Departamental de Salud, Gobernación del Cauca, Popayán, Colombia Gobernación del Cauca Popayán Colombia; 3 Departamento de Salud Pública, Facultad de Medicina, Universidad Nacional de Colombia, Bogotá, D.C., Colombia Universidad Nacional de Colombia Universidad Nacional de Colombia Bogotá, D.C. Colombia

**Keywords:** micología, tiña del cuero cabelludo, salud pública, mycology, tinea capitis, public health

## Abstract

**Introducción.:**

La tiña de la cabeza es una micosis que se presenta en el tejido queratinizado, afecta al cuero cabelludo y puede causar alopecia, prurito y descamación. Este tipo de micosis es más frecuente en niños de edad escolar, por lo que puede desencadenar un problema de salud pública. En Colombia, los principales agentes etiológicos reportados son los dermatofitos zoofílicos.

**Objetivo.:**

En el presente estudio se buscó caracterizar un brote de *tinea capitis* en 32 niños de un colegio de la zona rural del departamento del Cauca.

**Materiales y métodos.:**

Se llevó a cabo una investigación epidemiológica de campo en la que se aplicó una encuesta estructurada para caracterizar aspectos sociodemográficos y factores predisponentes para su ocurrencia. Se recolectaron muestras de escamas de cuero cabelludo y cabellos afectados para estudios micológicos. Finalmente, por medio de la Secretaría Departamental del Cauca y del hospital local, se manejó el brote de *tinea capitis* y se hicieron recomendaciones a los niños, los padres de familia y la población en general para prevenir estas micosis. Este estudio contó con el consentimiento informado verbal por parte de los padres de familia y los niños.

**Resultados.:**

El agente etiológico aislado en el 63 % de las muestras recolectadas fue *Trichophyton tonsurans* y el principal factor predisponente para esta micosis fue compartir máquinas rasuradoras (87,5 %). El agente etiológico de este brote de *tinea capitis* no inflamatoria fue un dermatofito antropofílico.

**Conclusión.:**

Idealmente, se deben practicar los estudios micológicos con el fin de establecer el agente etiológico y, así, plantear las terapéuticas y recomendaciones según las guías de manejo. Además, se debe realizar un trabajo multidisciplinario para el control del brote y la educación de la población respecto a esta micosis.

## Abstract (en)

**Introduction.:**

*Tinea capitis* is a mycosis of keratinized tissue, which affects the scalp and may cause alopecia, pruritus, and desquamation. This type of mycosis is more frequent in school-age children, and it may represent a public health problem; the main etiological agents reported for Colombia are zoophilic dermatophytes.

**Objective.:**

To characterize an outbreak of *Tinea capitis* in 32 children from a rural school in the department of Cauca.

**Materials and methods.:**

We conducted an epidemiological field study using a structured survey to characterize sociodemographic aspects and predisposing factors for this mycosis. We collected samples of affected scalp scales and hair for mycological studies. The children and the general population received recommendations, about these mycoses’ prevention, from Cauca’s health authorities and the local hospital. The parents verbally approved the informed consent.

**Results.:**

The etiological agent isolated in 63% of the collected samples was *Trichophyton tonsurans*, an anthropophilic dermatophyte, and the main predisposing factor was sharing razors (87.5%).

**Conclusions.:**

Ideally, mycological studies define the etiological agent to propose therapeutics and recommendations in agreement with management guidelines. Implementation of multidisciplinary measures to control the outbreak and educate the population is required.

Un brote es el aumento inusual del número de casos relacionados epidemiológicamente, aparece súbitamente y tiene diseminación localizada ^1^. Las micosis que afectan el cuero cabelludo son causadas por hongos filamentosos del grupo de los dermatofitos, que tienen la capacidad de degradar la queratina e infectar el estrato córneo, incluido el cabello.

Recientemente, los estudios filogenéticos de los dermatofitos, realizados mediante técnicas moleculares, han contribuido a su identificación y clasificación en siete clados: clado A, *Trichophyton*; clado B, *Epidermophyton*; clado C, *Nannizzia*; clado D, *Paraphyton*; clado E, *Lophophyton*; clado F, *Microsporum*, y clado G, *Arthroderma*^2^. La transmisión del hongo se produce por contacto directo o microtraumas con material queratinizado y contaminado con las formas infectivas del hongo (artroconidios). Esta puede darse de persona a persona, a partir de animales o del suelo, e indirectamente, mediante fómites como sombreros, cepillos para el cabello, etc. ^3^.

La *tinea capitis* es causada habitualmente por especies de *Microsporum*, *Nannizia* y *Trichophyton*. Existen cinco formas de presentación clínica, así como un estado de portador asintomático. La manifestación clínica más frecuente consiste en parches descamativos con alopecia -puede ser uno solo o múltiples- que es una característica común de la infección de tipo ectotrix (parasitismo externo); también, pueden variar de pocos a varios centímetros y se pueden asociar con eritema ^3^^-^^5^.

La segunda presentación frecuente consta de parches alopécicos con puntos negros visibles, típica de una infección de tipo endotrix (parasitismo interno). Estos puntos se ven negros porque la terminación distal del cabello se rompió en la superficie del cuero cabelludo, como resultado del debilitamiento del tallo del cabello, secundario a la infección endotrix. Los puntos negros se presentan en los folículos dentro de las áreas alopécicas, las cuales varían de tamaño -desde unos pocos centímetros a varios centímetros de diámetro- y pueden ser una sola lesión o múltiples lesiones ^4^^,^^5^.

La tercera forma clínica es una descamación generalizada con poca pérdida de cabello o sin existencia de zonas alopécicas evidentes, por lo que puede ser fácilmente confundida con una dermatitis seborreica ^4^^,^^5^.

El favo o tiña fávica (*tinea favus*) resulta de la infección por *Trycophyton schoenleinii* y rara vez por otros dermatofitos. Se presenta como un eritema perifolicular que progresa a la formación de costras amarillas, en forma de copa, llamadas escútulas, con un olor característicamente desagradable; son lesiones que podrían presentar coalescencia y formar masas adherentes alrededor de zonas alopécicas con inflamación intensa, con un posible proceso fibroso o cicatricial, asociado con la consecuente alopecia permanente ^6^.

El querión de Celso es la manifestación más seria de la *tinea capitis* y resulta de una importante reacción inmunológica contra la infección. Inicialmente, puede presentarse como una foliculitis supurativa, asociada con sensibilidad o dolor en la zona afectada, pero, luego, se forman placas pustulosas y costras gruesas supurativas, que en casos raros podrían asociarse con eritema nodoso ^7^.

Otros hallazgos en el examen físico son las adenopatías cervicales, especialmente con las presentaciones clínicas más graves, en las cuales también pueden coexistir reacciones eccematosas diseminadas o también llamadas reacciones de “autoeccematización”, secundarias a erupciones por dermatitis. Estas últimas consisten en una erupción eritematosa y pruriginosa generalizada, con pápulas que podrían comprometer la cabeza, el cuello, el tronco o las extremidades. Según su fisiopatología, se sugiere que es una reacción inmunológica a antígenos fúngicos o que podría corresponder a una reacción de hipersensibilidad tardía, posterior a la terapia antimicótica o incluso antes de ella ^4^^-^^7^.

Las infecciones de tiña de la cabeza se presentan con dos tipos de invasión capilar: la invasión de tipo ectotrix y la de tipo endotrix. La invasión de tipo ectotrix es comúnmente causada por *Microsporum canis*-dermatofito zoofílico- y *Nannizzia gypsea* -dermatofito geofílico-, los cuales desarrollan artroconidios, en forma de vaina, en la superficie del tallo del pelo, en donde la cutícula o base del pelo se destruye; en la tiña por *M. canis*, cuando el hongo es expuesto a la luz ultravioleta de la lámpara de Wood, se genera una fluorescencia verde-amarilla ^3^^-^^6^. La infección de tipo endotrix es causada por las especies antropofílicas *Trichophyton tonsurans* y *T. violaceum*, y se manifiesta con formación de artroconidios redondeados dentro del tallo del pelo; la cutícula no se destruye y la fluorescencia con luz de Wood es negativa. La evolución de este tipo de tiña es crónica y puede persistir hasta la edad adulta ^3^^-^^6^.

Las otras especies de dermatofitos zoofílicos, como *T. mentagrophytes* y *T. verrucosum*, suelen producir tiñas inflamatorias con presencia de pústulas, abscesos y supuración; presentación que se conoce como querión de Celso. Asimismo, puede haber inflamación, dolor y adenopatías regionales, el cabello se arranca con facilidad y puede dar lugar a una alopecia cicatricial ^3^^-^^7^.

En cuanto a su epidemiología, la *tinea capitis* afecta principalmente a los niños con edades entre los tres y los doce años, y está asociada con múltiples factores, como la falta de higiene personal, el hacinamiento, el bajo nivel socioeconómico, y las condiciones ambientales como el calor y la humedad ^8^.

La prevalencia de la *tinea capitis* varía notablemente alrededor del mundo, desde menos del 1 % en España hasta más del 50 % en países africanos como Nigeria, Etiopía y Ghana ^8^^-^^11^.

En Europa, el agente causal más común en humanos es *M. canis*, que se distribuye principalmente en el Mediterráneo y, también, en países como Austria, Hungría, Alemania y Polonia ^11^^,^^12^. La aparición de infecciones antropofílicas parece estar geográficamente restringida y se vincula a la inmigración desde países africanos ^11^. En el este y sur de África, el agente etiológico más común en humanos es *T. violaceum*, con una prevalencia que oscila entre el 56,7 y el 95 % ^10^^,^^11^. Sin embargo, en los últimos 10 a 20 años, la situación ha cambiado con la propagación de organismos como *T. tonsurans* en las Américas, Europa y África ^13^.

En el continente americano, Estados Unidos reporta que el 90 % de los casos de *tinea capitis* es causado por el dermatofito antropofílico *T. tonsurans* y se considera un trastorno endémico en escolares afroamericanos: 3 % presentan infección activa y 14 % son portadores asintomáticos ^8^^-^^14^.

En países como México, Perú y Chile, el agente etiológico asociado con la *tinea capitis* más frecuente es *M. canis*, seguido de *T. tonsurans*^14^^-^^17^. En lo que respecta a Colombia, las micosis no son de notificación obligatoria, por lo que se desconoce su frecuencia. En un estudio retrospectivo (1994-2013), realizado en un laboratorio de referencia de Colombia, con 415 pacientes con sospecha clínica de *tinea capitis*, se confirmaron 136 casos y se hicieron pruebas de laboratorio a 118. Se encontró que el principal agente etiológico fue *M. canis* en el 86 % (102/118) de los casos, seguido de *N. gypsea* en el 4 % (5/118) y *T. tonsurans* en el 3 % (4/118) ^17^. Sin embargo, no existen otros reportes que brinden más información sobre la prevalencia de *tinea capitis* en Colombia, lo que dificulta la actualización de los datos epidemiológicos en el país.

El presente estudio tuvo como objetivo caracterizar un brote de *tinea capitis* en un grupo de niños afrocolombianos de un colegio rural del departamento del Cauca, a partir de un caso índice. Se llevó a cabo por solicitud de la comunidad, de la secretaría de salud municipal y del personal del área de salud ambiental adscrito a la Secretaría Departamental de Salud del Cauca; y, por el interés del grupo investigador en identificar el agente etiológico, describir los factores asociados con su ocurrencia, y plantear estrategias de intervención y de educación que faciliten su control, en el marco de acciones de vigilancia epidemiológica ambiental, que puedan servir de base para el manejo de este tipo de brotes en Colombia.

## Presentación del caso

Se trata de un estudio de investigación de un brote de *tinea capitis*, en el que se utilizan métodos y técnicas diagnósticas ya aprobadas en el marco del sistema de salud y que hacen parte de las acciones de vigilancia epidemiológica de salud ambiental de la Secretaría Departamental de Salud del Cauca.

Se estableció el protocolo para el manejo de un brote, el cual se planteó a partir de un caso índice que llegó remitido por el personal del laboratorio rural al Laboratorio de Micología de la Facultad Ciencias de la Salud de la Universidad del Cauca, en septiembre de 2017.

**Figura 1:**
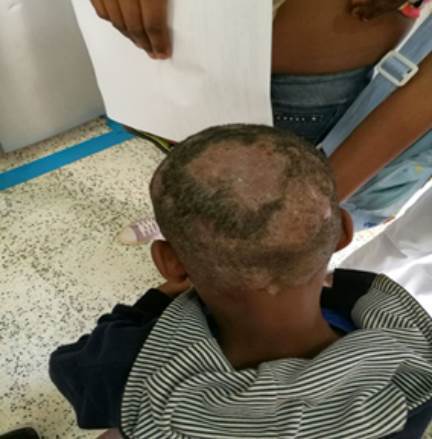
Niño, caso índice

El caso índice fue un niño, estudiante, de ocho años (figura 1), con sintomatología clínica indicativa de *tinea capitis* inflamatoria. El acudiente informó que los signos y síntomas llevaban tres años de evolución, sin diagnóstico micológico y que había recibido tratamientos esporádicos sin mejoría aparente; además, advirtió que, en la institución educativa del usuario ubicada en la zona rural del municipio de Suárez (Cauca), había más escolares con la misma sintomatología, lo que hizo sospechar un posible brote de *tinea capitis*.

Se llevó a cabo un trabajo de campo en la institución educativa con la participación del personal del área de salud ambiental de la Secretaría Departamental de Salud del departamento del Cauca y del Laboratorio de Microbiología de la Facultad de Salud de la Universidad del Cauca.

Por tratarse de un brote de interés en salud pública, se solicitó el consentimiento informado verbal a los padres de familia para aplicar una encuesta estructurada, e identificar los datos sociodemográficos y las condiciones predisponentes para la ocurrencia de la micosis. Se les explicó a los niños la técnica para recolectar las muestras clínicas de las lesiones y se les solicitó su permiso en forma verbal para seguir con el procedimiento.

**Figura 2:**
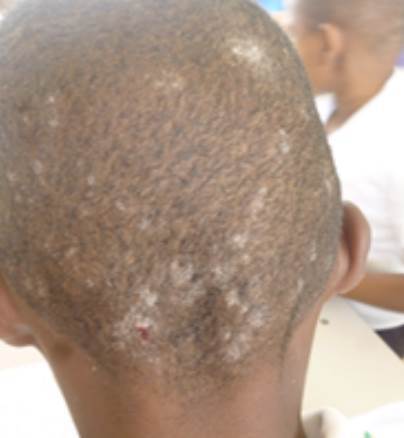
Paciente 8

Se recolectaron escamas del cuero cabelludo por raspado con hojas de bisturí y se cortaron cabellos de los 32 niños con signos y síntomas leves (figura 2). También, se recolectaron muestras de lesiones en proceso de recuperación del caso índice, quien se encontraba recibiendo tratamiento antimicótico con terbinafina. Se observó que dos de los niños presentaban máculas hipocrómicas en el tórax, por lo que se recolectaron muestras de esas lesiones con la técnica de la cinta engomada.

**Figura 3:**
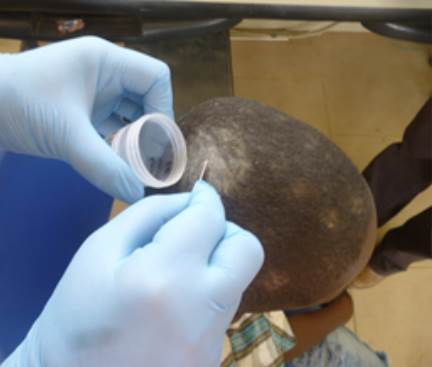
Recolección de muestras

**Figura 4:**
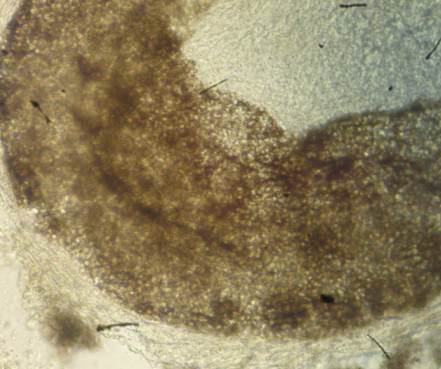
Parasitismo de tipo pilar, endotrix: las esporas y las hifas del hongo han invadido el interior de la estructura del pelo.

Las muestras clínicas fueron depositadas en frascos estériles pequeños (figura 3), trasladadas con las adecuadas medidas de bioseguridad y procesadas el mismo día en el Laboratorio de Microbiología. Se hizo el análisis microscópico, con solución de hidróxido de potasio al 40 % (figura 4), y siembras en medios agar Sabouraud, enriquecido con glucosa y que contenía cloranfenicol (0,05 mg/ml) (Oxoid, UK) y agar Mycosel™ (BBL, Becton Dickinson).

**Figura 5:**
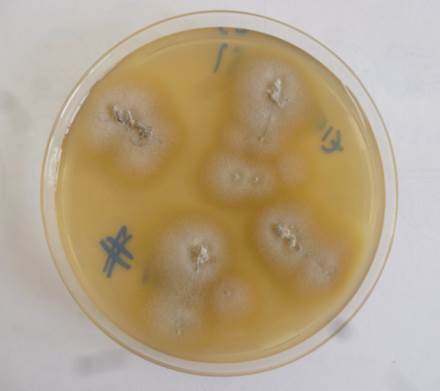
Cultivo de *Trichophyton tonsurans*

Los cultivos se incubaron a 28 °C hasta la observación de colonias de los 8 a los 15 días, en promedio. Las colonias se identificaron mediante la descripción macroscópica ^18^. En todos los cultivos positivos se observaron colonias de aspecto gamuzado, de color beige pálido, con ranuras radiales y, al reverso, de color marrón amarillento (figura 5).

**Figura 6:**
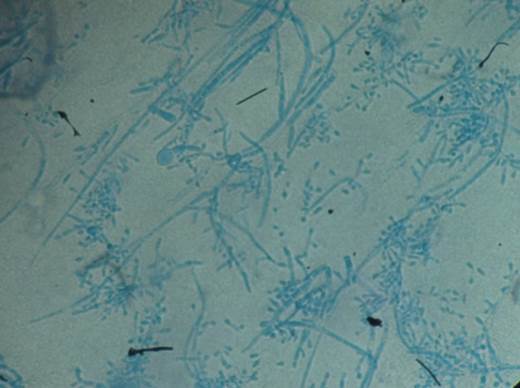
Montaje en azul de lactofenol: micromorfología de Trichophiton tonsurans a partir del microcultivo

Para la confirmación, se les practicó microcultivo, prueba de hidrólisis de la urea y prueba de perforación del cabello. A los cinco días de esporulación en el microcultivo, se observaron las características micromorfológicas de la conidiogénesis: hifas anchas irregulares, ramificadas, con numerosos tabiques, microconidios de varios tamaños y clamidosporas terminales (figura 6).

Las pruebas de hidrólisis de urea fueron positivas a los cinco días y las pruebas de perforación del cabello fueron positivas a los 14 días.

Se construyó una base de datos con la información recolectada en las encuestas y se hizo un análisis descriptivo utilizando el paquete estadístico SPSS™, versión 23 para Windows.

Para adelantar este estudio, se tuvieron en cuenta las consideraciones éticas pertinentes para salvaguardar el interés de la ciencia y el respeto de la privacidad de los participantes. Por tanto, los resultados se reportan de manera agrupada.

En cuanto a las características sociodemográficas de la población, la mayoría de los casos eran de sexo masculino 27/32 (84 %), con una media de edad de nueve años y datos extremos de 2 y 16 años. El nivel de escolaridad varió desde el grado de jardín hasta el séptimo. La *tinea capitis* fue más frecuente entre los niños del grado tercero de primaria en un 37,5 % (12/32), grupo en el que se encontraba el caso índice. Con respecto a la zona de residencia, la vivienda rural dispersa fue más frecuente, 65,6 % (21/32), que la residencia en el caserío de la vereda.

Como factor de riesgo epidemiológico, el más frecuente fue el uso compartido de máquinas rasuradoras en un 87,5 % (28/32). De estos, 15 niños frecuentaban a dos peluqueros que visitaban regularmente la zona veredal y 13 compartían la máquina rasuradora con sus familiares en casa (cuadro 1).

**Cuadro 1:** Condiciones que favorecen la transmisión de las micosis cutáneas en la población estudiada.

Categoría	Elementos o fómites	N=32	Porcentaje
Comparten elementos de uso personal o prendas de vestir	Máquina rasuradora	28	87,5
	Toallas	18	56,3
	Cepillos o peines	16	50,0
	Gorras	15	46,9
Mascotas en casa	Tienen mascotas (perros y gatos)	30	93,8
	Permiten que la mascota se suba a los muebles	3	9,4
Comparten cama	Con una persona	13	40,6
	Con dos o más personas	8	25,0

En el examen físico, se encontraron 32 niños con lesiones alopécicas, descamativas, no inflamatorias, en punto negro. Dos de ellos presentaban lesiones en el tórax, indicativas de pitiriasis versicolor. La observación microscópica de las muestras de escamas de estas lesiones torácicas resultó positiva para *tinea capitis*. Las 32 muestras de cabellos se analizaron mediante microscopía, previo tratamiento con preparación de hidróxido de potasio (KOH al 40 %) y, en el 78 % (26/32), se detectó parasitismo pilar de tipo endotrix tricofítico (figura 4). De estos, cinco cultivos fueron negativos, mientras que, en los restantes, se aisló e identificó *T. tonsurans* (63 %; 21/32).

Cabe destacar que, al momento de la toma de las muestras, cinco de los escolares habían recibido tratamientos empíricos de forma intermitente: dos con clotrimazol (6,3 %), dos con terbinafina (6,3 %) y uno con una combinación de clotrimazol más un corticosteroide (3,1 %). Los estudios micológicos del caso índice, que ya estaba en tratamiento, resultaron negativos.

Una vez se identificó el agente etiológico, se remitió el reporte de laboratorio a la oficina de Salud Ambiental de la Secretaría Departamental de Salud, encargada de contactar a la Entidad Promotora de Salud de los niños para acceder a los tratamientos antimicóticos. Igualmente, los resultados se enviaron al responsable del programa de salud ambiental del Hospital de Suárez (Cauca) para el manejo del brote epidémico de *tinea capitis* y demás actividades relacionadas con promoción de la salud y prevención de la enfermedad, como charlas informativas en la escuela para los niños, profesores, familias y los peluqueros de la zona.

Pasados seis meses, se investigó si los niños habían recibido tratamiento y, desde el hospital del municipio de Suárez (Cauca), se informó que tan solo tres niños (9,4 %) recibieron tratamiento completo, ocho (25 %) tratamiento incompleto y 21 de ellos (65 %) no recibieron ningún tratamiento.

Es de aclarar que no se tomaron muestras clínicas para estudios micológicos para demostrar la resolución de la micosis en los pacientes.

### Consideraciones éticas

Como parte de las acciones de vigilancia epidemiológica de salud ambiental de la Secretaría Departamental de Salud del Cauca, se estableció el protocolo para el manejo de un brote en una escuela rural y se solicitó consentimiento informado verbal a los padres de familia y a los niños que tenían signos y síntomas de la micosis cutánea en el cuero cabelludo.

## Discusión

En el presente estudio se pudo observar, por sus características, que los 32 niños presentaban un cuadro clínico sugestivo de *tinea capitis* seca. En el examen directo, por microscopía, se detectó parasitismo pilar de tipo endotrix tricofítico en el 78 % (25/32) de los casos. Esto indica que solo en el interior del cabello se encontraron artroconidios del hongo, con un diámetro superior a 8 µm, sin destrucción de la cutícula y congruente con el tipo de parasitismo pilar que desarrolla *T. tonsurans*^18^^-^^20^.

La sensibilidad del examen directo varía según la experiencia del observador y la capacitación de la persona que toma la muestra, de quien dependen su cantidad y calidad. Habitualmente, el examen directo se efectúa en fresco utilizando sustancias que favorecen la disgregación de la queratina y aclaran la preparación, como el clásico hidróxido de potasio. Estas sustancias facilitan la visualización de las estructuras micóticas (hifas, conidios o blastoconidias) por su alto índice de refracción ^18^^,^^19^. Asimismo, los cultivos suelen tener una sensibilidad de más del 90 % cuando los pacientes no han recibido tratamientos y se considera la prueba de referencia para el diagnóstico definitivo ^18^^,^^19^.

En este caso, por el tipo de colonias, las pruebas confirmatorias y el desarrollo de parasitismo pilar de tipo endotrix, se confirmó que el hongo aislado en todos los cultivos era *T. tonsurans*. Se encontró una gran concordancia entre los cultivos positivos y la observación del tipo de parasitismo pilar al examen microscópico, en una relación de 1,2:1.

En este trabajo, se evidenció que cinco de los niños ya tenían tratamientos parciales en forma empírica con antimicóticos, los demás con resultados negativos pudieron haber tenido una carga baja o nula de esporas del hongo, lo que no permitió su detección en los cultivos.

Este brote de *tinea capitis* afectó más frecuentemente a la población infantil masculina, similar a lo que indican algunas revisiones sobre el tema ^13^, trabajos de investigación y otros brotes de *tinea capitis*, en países como España ^9^, Chile ^16^, Paraguay ^21^, Turquía ^22^, India y Egipto ^23^. Sin embargo, un estudio realizado en Guatemala indicó que el sexo femenino fue el más afectado ^24^. Algunas investigaciones indican que los hombres tienen una mayor predisposición a la tiña del cuero cabelludo y se postula que está parcialmente relacionada con una longitud de cabello más corta. Además, pueden quedar como portadores asintomáticos, constituyéndose en un reservorio persistente ^25^.

En el presente estudio, se identificó *T. tonsurans* como único agente etiológico del brote de *tinea capitis* en un grupo de escolares afrodescendientes, similar a lo reportado en Norteamérica y en países de la costa del Caribe, como República Dominicana y Puerto Rico ^26^^,^^27^.

No hay una explicación clara sobre la predilección de *T. tonsurans* por la población afrodescendiente ^9^. Se conoce que, en esta población, las características del folículo y el tallo piloso son curvados o en espiral, y su disposición paralela a la superficie cutánea hacen que el pelo sea más frágil y quebradizo, lo que podría sugerir mayor vulnerabilidad a enfermedades cutáneas asociadas con discromías, hiperpigmentación o hipopigmentación ^28^^,^^29^.

Un estudio en población infantil con *tinea capitis* por *T. tonsurans* en Estados Unidos, indicó que hay genes implicados en la propensión a estas infecciones cutáneas en la población afroamericana ^30^. Recientemente, se reportó una predilección genética de sufrir dermatofitosis relacionada con la deficiencia de la proteína de tipo caspasa CARD9, la expresión de moléculas del complejo mayor de histocompatibilidad HLA-DR4 y HLA-DR8, la baja expresión de la interleucina 22, β-defensina 2 y 4, así como mutaciones en la dectina 1, lo que aumenta la prevalencia de la enfermedad en miembros de una misma familia ^31^. Además, las migraciones de poblaciones influyen en la distribución de la enfermedad, lo que ha ocasionado un cambio en su perfil epidemiológico ^9^^,^^12^^,^^16^^,^^23^^,^^32^^,^^33^

*Trychophyton tonsurans* es un dermatofito antropofílico y un agente patógeno oportunista, considerado como especie emergente en Colombia ^34^. Causa brotes epidémicos en escuelas, hogares infantiles e intrafamiliares donde se mantiene contacto cercano con compañeros y familiares, y en los que prevalece el uso de elementos comunes como las máquinas rasuradoras de este caso, en el que los niños frecuentaban dos peluqueros que atendían en la misma vereda o compartían la misma máquina rasuradora en la familia.

Este sería el segundo registro de un brote de *tinea capitis* por *T. tonsurans* en Colombia desde que, en 1981, Castañeda *et al*. reportaron un brote epidémico en cuatro niños de una aldea para huérfanos en Bogotá ^35^. Otros brotes en población escolar y causados por este mismo agente etiológico, se han reportado en Valparaíso (Chile) ^16^, en Madrid (España) ^36^ y en París (Francia) ^37^.

Debe considerarse que el municipio de Suárez se encuentra ubicado al noroccidente del departamento del Cauca, con una altura de 1.081 msnm y una temperatura media de 27 °C ^38^, condiciones que determinan un ambiente cálido y húmedo, favorable para el desarrollo de hongos. A esto se suman factores predisponentes, como la edad de los pacientes, las condiciones sociodemográficas, la falta de agua potable en las casas, la deficiencia del aseo personal diario y la costumbre de compartir objetos de aseo personal, como las máquinas rasuradoras, los cepillos de cabello y las gorras, los cuales permiten la contaminación con los artroconidios de *T. tonsurans*. Se debe hacer una descontaminación previa de los fómites para su uso entre persona y persona, situación ya identificada en otros países, donde se asocia la presentación de esta micosis con deficiencias en el cuidado del aseo personal, los objetos personales compartidos y los estratos socioeconómicos bajos ^3^^,^^32^^-^^33^.

*Trychophyton tonsurans* es muy contagioso y puede causar infecciones graves y crónicas ^39^, como sucedió en el caso índice, el niño que llevaba más de tres años con los síntomas y en quien se observó parasitismo pilar de tipo endotrix, pero no se logró aislar el hongo por encontrarse, en ese momento, en tratamiento antimicótico.

Por lo tanto, es importante establecer medidas educativas encaminadas a disminuir la frecuencia y la prevalencia de esta dermatofitosis, así como a prevenir su transmisión y sus recidivas en la población general, en especial, en la población más afectada que son los niños en edad escolar ^40^.

Actualmente, se recomienda el estudio clínico y el de laboratorio: la sensibilidad disminuida o la resistencia *in vitro* de los dermatofitos a los antimicóticos de uso común (terbinafina, itraconazol, voriconazol y amorolfina), y la resistencia del paciente, ya que el tratamiento de estas afecciones no depende únicamente de la concentración inhibitoria mínima del hongo, sino también, de la reacción del huésped ^41^^-^^45^.

En cuanto a la prevención, debe considerarse que los artroconidios de los dermatofitos pueden ser viables por meses o años en el medio ambiente, por lo que se recomienda hacer la desinfección regular de fómites como cepillos, peines y máquinas de peluquería con una solución acuosa de hipoclorito de sodio al 2 %; además, se debe remover todo el material que contenga queratina, como piel muerta y pelos, lo que facilita la desinfección. Cuando sea posible, se debe asear con aspiradora todos los posibles fómites en el hogar, como sillas y otros muebles ^40^. Como el 50 % de los miembros de la familia de un caso pueden verse afectados en forma asintomática, se recomienda hacer búsqueda activa entre todos ellos y, por supuesto, tratar los que resulten positivos.

En portadores asintomáticos con una gran carga de esporas, la terapia oral suele justificarse. Si la carga de esporas es poca, los hongos pueden erradicarse con tratamiento tópico único, pero deben hacerse un seguimiento cercano y estudios de micología repetidos, para asegurarse de que el tratamiento haya sido eficaz. Idealmente, la erradicación fúngica, completa y eficaz, en los portadores asintomáticos, requiere del apoyo y la participación de los trabajadores sanitarios comunitarios, incluidas las enfermeras ^40^.

Como conclusión, se determinó que el agente etiológico del brote de *tinea capitis* no inflamatoria en el grupo de estudiantes del área rural del municipio de Suárez fue *T. tonsurans*. Se identificaron condiciones epidemiológicas predisponentes para la transmisión del hongo, principalmente, el uso compartido de elementos como las rasuradoras y la deficiencia en la higiene personal.

Es importante resaltar que, antes de iniciar un tratamiento farmacológico, sistémico o tópico, se deberían practicar las respectivas pruebas de laboratorio para identificar el agente etiológico de la infección y, así, tener una aproximación diagnóstica más acertada y elegir la mejor alternativa terapéutica. Se requiere confirmar la curación de la micosis, para lo cual es necesario hacer seguimiento a los niños después del tratamiento. El tratamiento para la infección debe ser sistémico, con la coadyuvancia de medicamentos tópicos en las áreas afectadas.

En cuanto a la ocurrencia de brotes epidémicos, se requiere una intervención multidisciplinaria para su control, con acciones que involucren a profesionales de la salud (asistenciales y de salud ambiental), visita inmediata al sitio del brote, recolección de muestras clínicas, estudios micológicos y, finalmente, educación sobre la prevención de la micosis a la población involucrada, familiares, instituciones educativas y agentes responsables de las salas de peluquería.
